# 14-3-3σ regulation of and interaction with YAP1 in acquired gemcitabine resistance via promoting ribonucleotide reductase expression

**DOI:** 10.18632/oncotarget.7394

**Published:** 2016-02-15

**Authors:** Li Qin, Zizheng Dong, Jian-Ting Zhang

**Affiliations:** ^1^ Department of Pharmacology and Toxicology and IU Simon Cancer Center, Indiana University School of Medicine, Indianapolis, Indiana, USA

**Keywords:** 14-3-3σ, gemcitabine resistance, ribonucleotide reductase, YAP1, gene expression

## Abstract

Gemcitabine is an important anticancer therapeutics approved for treatment of several human cancers including locally advanced or metastatic pancreatic ductal adenocarcinoma (PDAC). Its clinical effectiveness, however, is hindered by existence of intrinsic and development of acquired resistances. Previously, it was found that 14-3-3σ expression associates with poor clinical outcome of PDAC patients. It was also found that 14-3-3σ expression is up-regulated in gemcitabine resistant PDAC cells and contributes to the acquired gemcitabine resistance. In this study, we investigated the molecular mechanism of 14-3-3σ function in gemcitabine resistance and found that 14-3-3σ up-regulates YAP1 expression and then binds to YAP1 to inhibit gemcitabine-induced caspase 8 activation and apoptosis. 14-3-3σ association with YAP1 up-regulates the expression of ribonucleotide reductase M1 and M2, which may mediate 14-3-3σ/YAP1 function in the acquired gemcitabine resistance. These findings suggest a possible role of YAP1 signaling in gemcitabine resistance.

## INTRODUCTION

Gemcitabine is a deoxycytidine analogue approved as the first-line chemotherapeutic drug for patients with locally advanced or metastatic pancreatic ductal adenocarcinoma (PDAC) [1, 2], one of the cancers having poorest prognosis with 5-year survival rate hovering only around 7% [3]. One of the major causes for the poor prognosis of PDAC is that it is intrinsically resistant or can acquire resistance to treatments including gemcitabine. Several molecular mechanisms of gemcitabine resistance have been identified including dysregulation of enzymes participating in gemcitabine metabolism, down-regulation of gemcitabine importer hENT1, down-regulation of rate-limiting enzyme dCK, and up-regulation of gemcitabine target, ribonucleotide reductase M1 and M2 (RRM1 and RRM2) [4]. RRM has also been suggested to mediate C-MYC-dependent suppression of senescence [5].

It has also been shown previously that increased 14-3-3σ expression associates with poor prognosis of PDAC [6, 7]. Recently, using a newly established gemcitabine resistant PDAC cell line, G3K derived from MiaPaCa-2 cells via stepwise gemcitabine selections, we showed that 14-3-3σ expression is upregulated via epigenetic regulation and contributes to the acquired gemcitabine resistance in the gemcitabine-selected PDAC G3K cells [8]. In addition to the acquired gemcitabine resistance in PDAC cells, 14-3-3σ has been implicated in acquired doxorubicin resistance in breast cancer cells [9] and in cisplatin resistance of colon cancer cells [10]. Although 14-3-3σ is known to belong to the highly conserved 14-3-3 protein family that binds to many phosphoserine/phosphothreonine proteins important in multiple biological processes such as signal transduction, cell cycle control, and survival [11-14], how its increased expression contributes to acquired drug resistance in general and gemcitabine resistance more specifically is largely unknown.

One of the phosphoserine/phosphothreonine protein partners of 14-3-3 family proteins is YAP1, a transcriptional coactivator in the Hippo/YAP pathway, which binds to and activates several transcription factors including Runx [15] and the highly conserved TEAD/TEF transcription factors [16]. Once phosphorylated at a key serine residue (Ser^127^), YAP is sequestered in the cytoplasm by binding to 14-3-3 proteins, where it can no longer function to promote target gene expression [17]. The crystal structure of 14-3-3σ/YAP1 phosphopeptide complex has been resolved at 1.15Å resolution, suggesting the interaction between YAP1 and 14-3-3σ [18]. In this study, we show that 14-3-3σ not only interacts with YAP1 but also regulates YAP1 expression. The increased 14-3-3σ and YAP1 expression cooperates to contribute to the acquired gemcitabine resistance by inhibiting gemcitabine-induced caspase 8 activation possibly via up-regulating the expression of gemcitabine target protein RRM1 and RRM2.

## RESULTS

### YAP1 over-expression in G3K cells and its regulation by 14-3-3σ

**Figure 1:**
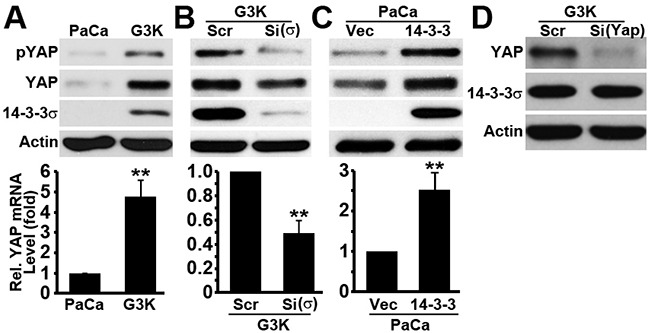
Expression and regulation of YAP1 **A.** Relative expression of YAP1 and pYAP1 in the parental MiaPaCa-2 and the gemcitabine-resistant derivative G3K cells. **B–C.** Effect of 14-3-3σ knockdown in G3K cells (B) or ectopic over-expression in MiaPaCa-2 cells (C) on YAP1 and pYAP1 expression. **D.** Effect of YAP1 knockdown on 14-3-3σ expression. Actin was used as a loading control for Western blot (upper panels) and GAPDH was used as an internal control for real-time RT-PCR (lower panels). (n=3, **p<0.01)

To determine the potential role of YAP1 in 14-3-3σ-mediated gemcitabine resistance, we first tested the level of total YAP1 and pYAP1 (Ser^127^ phosphorylated) in the parental MiaPaCa-2 and its gemcitabine resistant derivative G3K cells. As shown in Figure 1A, both YAP1 and pYAP1 proteins as well as YAP1 mRNA were significantly increased in G3K compared with MiaPaCa-2 cells. Because 14-3-3σ expression is also up-regulated in G3K cells (Figure 1A), we next investigated if 14-3-3σ and YAP1 expression is related by first knocking down 14-3-3σ in G3K cells and testing its effect on YAP1 expression. Figure 1B shows that 14-3-3σ knockdown significantly reduced YAP1 mRNA and protein levels. Ectopically over-expressing 14-3-3σ in the parental MiaPaCa-2 cells increased YAP1 mRNA and protein levels (Figure 1C). However, knocking down YAP1 had no effect on 14-3-3σ expression (Figure 1D). These findings suggest that 14-3-3σ may regulate YAP1 expression but not *vice versa*.

### Role of YAP1 in gemcitabine resistance

**Figure 2:**
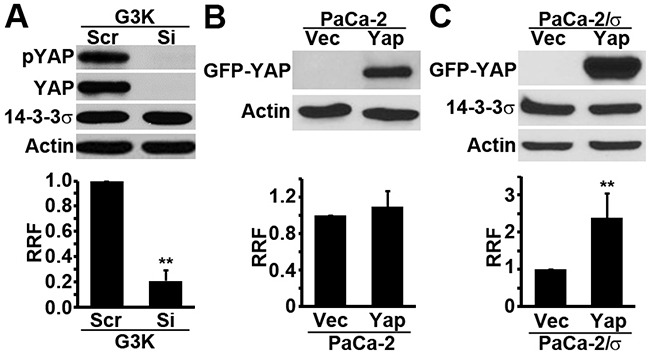
Role of YAP1 in gemcitabine resistance YAP1 siRNA was transiently transfected into G3K cells **A.** and GFP-YAP1 cDNA was transiently transfected into MiaPaCa-2 cells **B.** or MiaPaCa-2 cells with stable 14-3-3σ expression (PaCa-2/σ) **C.** followed by Western blot analysis of YAP1 expression (upper panels) and survival analysis in the presence of gemcitabine using MTT assay (lower panels). RRF=relative resistance factor. Actin was used as a loading control for Western blot. (n=4, **p<0.01).

To determine if the increased YAP1 expression in G3K cells potentially contributes to the acquired gemcitabine resistance, we first knocked down YAP1 in G3K cells followed by examining the difference in gemcitabine resistance using MTT assay. As shown in Figure 2A, YAP1 knockdown was successfully achieved without affecting 14-3-3σ expression and it dramatically reduced gemcitabine resistance of G3K cells. Unlike 14-3-3σ over-expression in the parental MiaPaCa-2 cells, which resulted in increased gemcitabine resistance [8], ectopic over-expression of GFP-YAP1 in MiaPaCa-2 cells did not significantly influence the gemcitabine response (Figure 2B). This observation is intriguing and inconsistent with our findings of YAP1 knockdown in G3K cells (Figure 2A).

Because YAP1 does not affect 14-3-3σ expression and the parental MiaPaCa-2 cells express little or no 14-3-3σ, YAP1 over-expression alone in the parental MiaPaCa-2 cells may not be able to induce gemcitabine resistance if it works together with 14-3-3σ. To test this possibility, we took advantage of MiaPaCa-2 cells with stable over-expression of Flag-14-3-3σ (MiaPaCa-2/σ cells) and over-expressed GFP-YAP1 followed by survival assay in the presence of gemcitabine. As shown in Figure 2C, over-expression of GFP-YAP1 significantly increased gemcitabine resistance of the MiaPaCa-2/σ cells. Thus, it is possible that YAP1 up-regulation contributes to the acquired gemcitabine resistance in G3K cells but it may require the presence of 14-3-3σ for this function.

### 14-3-3σ and YAP1 are inter-dependent in gemcitabine resistance

**Figure 3:**
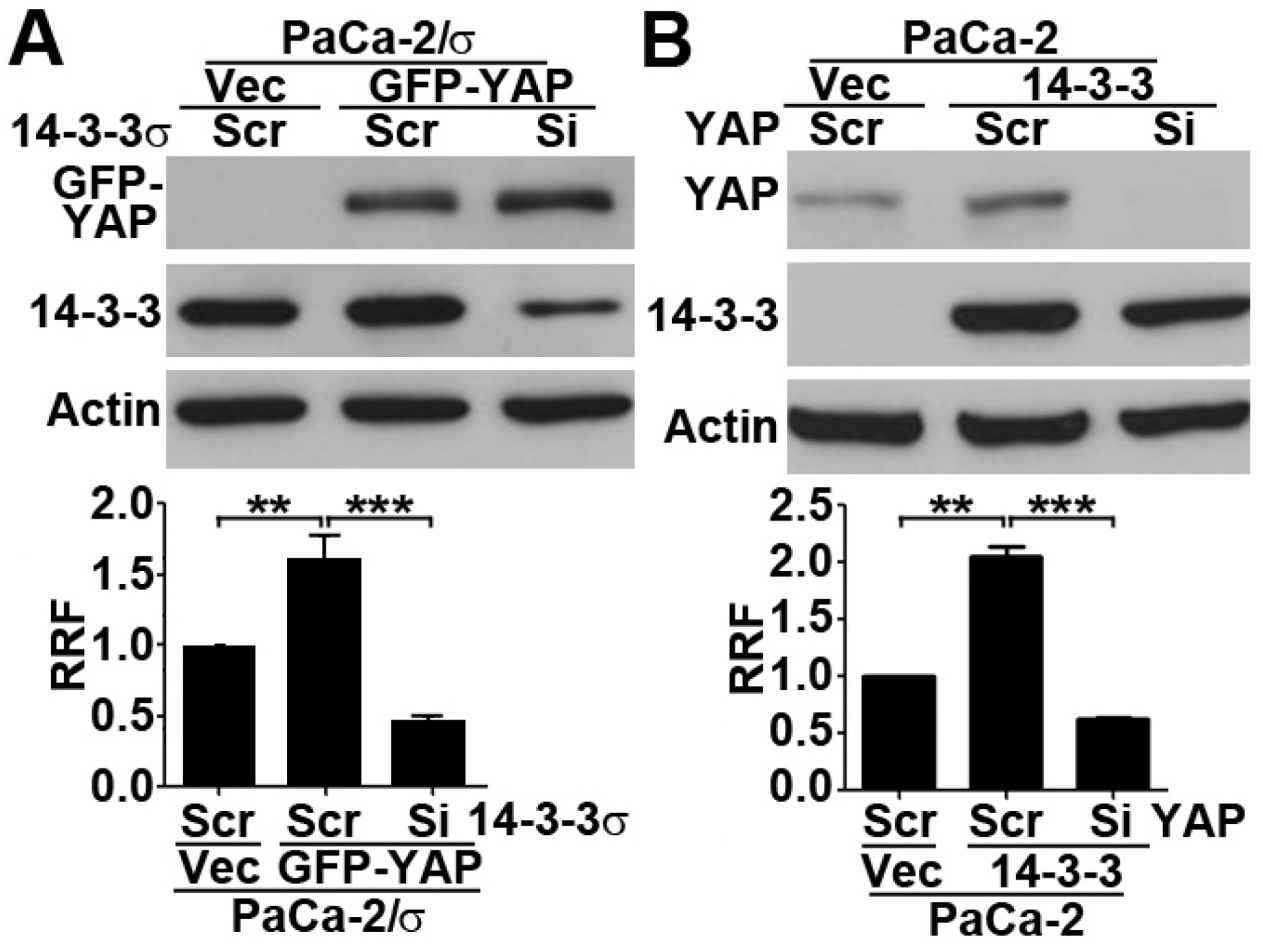
14-3-3σ and YAP1 are inter-dependent in gemcitabine resistance **A.** Effect of 14-3-3σ knockdown on gemcitabine resistance in MiaPaCa-2 cells with stable over-expression of 14-3-3σ and with YAP1 over-expression. **B.** Effect of YAP1 knockdown on gemcitabine resistance in MiaPaCa-2 cells with 14-3-3σ over-expression. Actin was used as loading control for Western blot analysis (upper panels). Lower panels show relative resistance factor (RRF) derived from dose-response curves from MTT assays. (n=3, **p<0.01, ***p<0.001)

To further investigate if YAP1 requires 14-3-3σ in gemcitabine resistance, we transiently over-expressed GFP-YAP1 in MiaPaCa-2/σ cells followed by knocking down 14-3-3σ and survival assay. As shown in Figure 3A, GFP-YAP1 over-expression significantly increased gemcitabine resistance of MiaPaCa-2/σ cells as expected. However, knocking down 14-3-3σ in MiaPaCa-2/σ cells with GFP-YAP1 over-expression completely eliminated the GFP-YAP1 over-expression-induced increase in gemcitabine resistance. It is noteworthy that the gemcitabine resistance of the 14-3-3σ knockdown cells is significantly lower than the control MiaPaCa-2/σ cells, demonstrating that the basal resistance induced by 14-3-3σ over-expression in MiaPaCa-2/σ cells is reduced by 14-3-3σ knockdown. This observation is consistent with our previous findings demonstrating the role of 14-3-3σ in gemcitabine resistance [8]. Similarly, YAP1 knockdown in MiaPaCa-2/σ cells also eliminated 14-3-3σ-induced gemcitabine resistance (Figure 3B). These findings suggest that YAP1 and 14-3-3σ may depend on each other for their functions in gemcitabine resistance.

**Figure 4:**
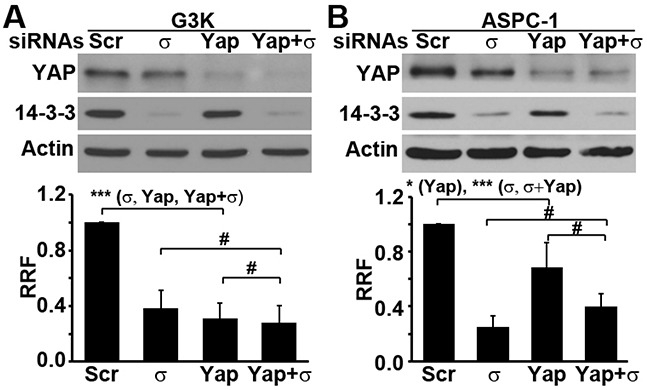
Both 14-3-3σ and YAP1 are required for their role in gemcitabine resistance G3K cells **A.** or ASPC-1 cells **B.** were transiently transfected with either scrambled (Scr) control siRNA, 14-3-3σ siRNA, and YAP1 siRNA alone, or co-transfected with both 14-3-3σ and YAP1 siRNA simultaneously, followed by Western blot analysis of YAP1, 14-3-3σ and actin loading control or MTT assay in the presence of gemcitabine for analysis of gemcitabine resistance. (n=3-5, *p<0.05, **p<0.01, ***p<0.001, #p>0.05). Actin was used as a loading control for Western blot.

To better address this hypothesis, we performed double knockdown experiments in G3K cells. As shown in Figure 4A, YAP1 and 14-3-3σ could be simultaneously knocked down. While knocking down 14-3-3σ or YAP1 individually significantly reduced gemcitabine resistance of G3K cells, which is consistent with above findings, the double knockdown of 14-3-3σ and YAP1 did not result in further reduction in gemcitabine resistance. It is also noteworthy that the double knockdown did not affect the proliferation of G3K cells (data not shown). To ensure the above observation on gemcitabine resistance is not due to the use of a specific gemcitabine-selected cell line G3K, we performed a similar experiment using another PDAC cell line, ASPC-1, that expresses endogenous 14-3-3σ. As shown in Figure 4B, individual knockdowns of 14-3-3σ or YAP1 resulted in significant reduction in gemcitabine resistance in ASPC-1 cells. Similar to G3K cells, double knockdown did not result in further reduction in gemcitabine resistance in ASPC-1 cells. Hence, 14-3-3σ and YAP1 may cooperate and require each other in pancreatic cancer cell survival against gemcitabine treatment.

### 14-3-3σ and YAP1 co-localize and interact with each other

**Figure 5:**
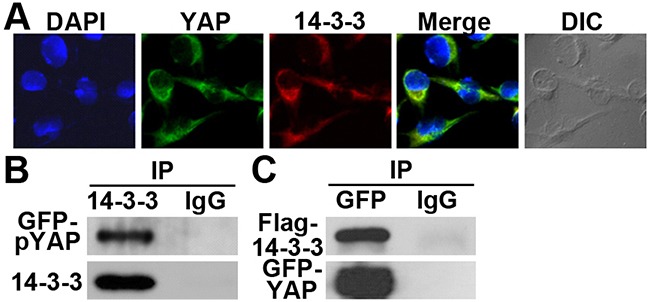
14-3-3σ co-localization and interaction with YAP1 **A.** Co-localization of 14-3-3σ and YAP1 in G3K cells viewed using a confocal microscopy. **B–C.** Co-immunoprecipitation of 14-3-3σ and YAP1 in G3K cells. The ectopic GFP-YAP1 alone (B) or together with Flag-14-3-3σ (C) were over-expressed in G3K cells followed by immunoprecipitation with 14-3-3σ (B) or GFP (C) antibody or control normal IgG and Western blot analysis of pYAP1 or total YAP1 and 14-3-3σ.

To further test the cooperativity between 14-3-3σ and YAP1, we tested if they co-localize and possibly interact with each other. As shown in Figure 5A, both YAP1 and 14-3-3σ co-localized in the cytoplasm of G3K cells. We next performed co-immunoprecipitation analysis of 14-3-3σ and GFP-YAP1. As shown in Figure 5B, immunoprecipitation of 14-3-3σ resulted in co-precipitation of the phosphorylated YAP1. Immunoprecipitation of YAP1 also resulted in co-precipitation of the ectopically over-expressed Flag-14-3-3σ in G3K cells. Thus, 14-3-3σ likely can bind to and form a complex with pYAP1.

### 14-3-3σ and YAP1 protect against gemcitabine-induced caspase-8 activation and apoptosis

**Figure 6:**
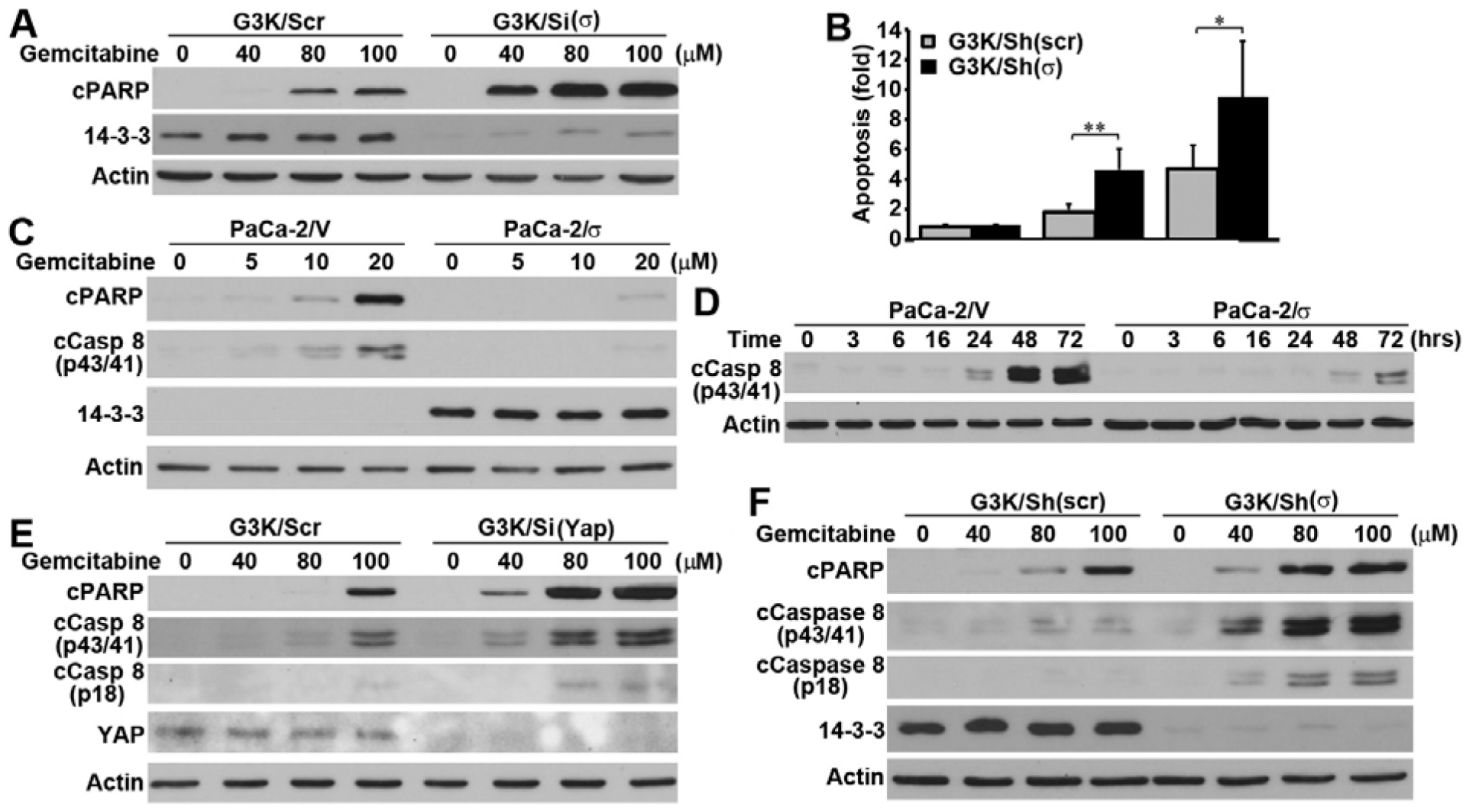
Role of 14-3-3σ and YAP1 in gemcitabine-induced apoptosis and caspase-8 activation **A–B.** Effect of 14-3-3σ knockdown on gemcitabine-induced apoptosis in G3K cells by determination of PARP1 cleavage using Western blot analysis (A) and using Cell Death Detection ELISA (B). (N=n, *p<0.05, **p<0.001). **C–D.** Effect of 14-3-3σ over-expression on gemcitabine-induced PARP1 cleavage and caspase 8 activation. **E–F.** Effect of YAP1 (E) and 14-3-3σ (F) knockdown on gemcitabine-induced PARP1 cleavage and caspase 8 activation.

To understand the mechanism of 14-3-3σ and YAP1-induced gemcitabine resistance, we tested if they protect PDAC cells against gemcitabine-induced apoptosis. As shown in Figure 6A, 14-3-3σ knockdown in G3K cells led to dose-dependent increase in gemcitabine-induced PARP-1 cleavage, an indicator of apoptosis. To confirm this finding and to ensure the effect was not due to off-target effect of the siRNA used, we performed apoptosis assay using the Cell Death Detection ELISA kit that quantifies the level of DNA fragmentation in a stable 14-3-3σ knockdown clone of G3K cells [8]. Figure 6B shows that the stable 14-3-3σ knockdown using shRNA with a different targeting sequence from the siRNA used in the above experiments also significantly increased gemcitabine-induced apoptosis of G3K cells. Meanwhile, 14-3-3σ over-expression in the parental MiaPaCa-2 cells eliminated gemcitabine-induced PARP1 cleavage (Figure 6C–6D). Similar to 14-3-3σ knockdown, YAP1 knockdown in G3K cells also led to dose-dependent increase in gemcitabine-induced PARP1 cleavage (Figure 6E). Thus, both YAP1 and 14-3-3σ helps protect PDAC cells against gemcitabine-induced apoptosis.

To investigate the specific apoptotic pathway that 14-3-3σ and YAP1 interferes, we first analyzed caspase-8 and caspase-9 activation following gemcitabine treatment in G3K cells. As shown in Figure 6, gemcitabine-induced caspase-8 cleavage and activation was dramatically elevated in YAP1 (Figure 6E) and 14-3-3σ (Figure 6F) knockdown G3K cells and dramatically reduced in the 14-3-3σ-over-expressing MiaPaCa-2 cells (Figure 6C) compared with their respective control cells. However, caspase-9 activation was not affected (data not shown). It is noteworthy that G3K cells with stable 14-3-3σ knockdown using shRNA (Figure 6F) with different targeting sequence from siRNA (Figure 6A) also caused similar increase in gemcitabine-induced PARP1 cleavage, consistent with the findings shown in Figure 6B. Thus, likely 14-3-3σ and YAP1 may protect against gemcitabine-induced apoptosis by attenuating gemcitabine-induced caspase-8 activation.

### Role of 14-3-3σ and YAP1 in regulating RRM1 and RRM2 expression

**Figure 7:**
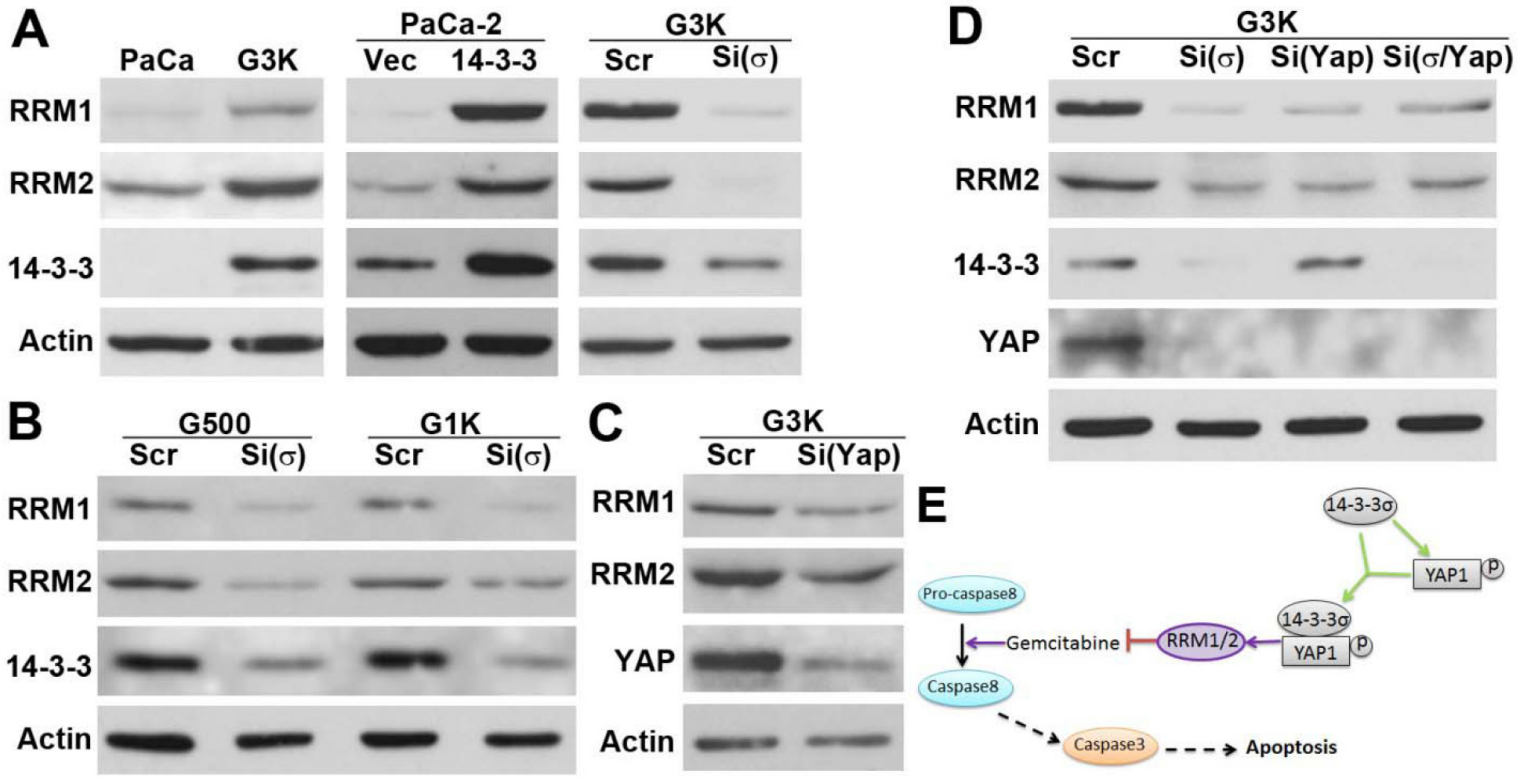
Regulation of RRM1 and RRM2 expression by 14-3-3σ/YAP1 **A.** Western blot analysis of RRM1 and RRM2 expression in the parental MiaPaCa-2 and gemcitabine resistant G3K cells and following 14-3-3σ over-expression in MiaPaCa-2 cells or 14-3-3σ knockdown in G3K cells. **B.** Effect of 14-3-3σ knockdown on RRM1 and RRM2 expression in the intermediate resistant cell line G500 and G1K cells derived during stepwise selection of G3K cells. **C.** Effect of YAP1 knockdown on RRM1 and RRM2 expression in G3K cells. **D.** Effect of knocking down 14-3-3σ and YAP1 individually or both simultaneously on RRM1 and RRM2 expression in G3K cells. **E.** Schematic model of 14-3-3σ regulation and interaction with YAP1 in gemcitabine resistance.

To understand how 14-3-3σ and YAP1 interfere with gemcitabine-induced apoptosis in PDAC cells, we resorted to the previously well known mechanisms of gemcitabine resistance, namely the over-expression of ribonucleotide reductase M1 and M2 (RRM1 and RRM2) [19, 20]. We have shown previously that the gemcitabine-resistant G3K cells also over-express RRM1 and RRM2 [8] (see also Figure 7A). To determine if there is a potential relationship between RRM1/RRM2 and 14-3-3σ/YAP1, we first tested RRM1 and RRM2 expression in MiaPaCa-2 cells with stable 14-3-3σ over-expression. As shown in Figure 7A, both RRM1 and RRM2 are up-regulated by 14-3-3σ over-expression and down-regulated by 14-3-3σ knockdown using siRNA. Similarly, stable 14-3-3σ knockdown in G3K cells using shRNA also reduced expression of RRM1 and RRM2 (data not shown). Furthermore, 14-3-3σ knockdown also reduced the expression of both RRM1 and RRM2 in G500 and G1K cells with intermediate gemcitabine resistance, which were produced during selection of G3K cells [8], suggesting that 14-3-3σ regulation of RRM1 and RRM2 expression is not specific to G3K cells. Similar to 14-3-3σ knockdown, YAP1 knockdown also reduced the expression of both RRM1 and RRM2 in G3K cells (Figure 7C). Thus, RRM1 and RRM2 may be the downstream mediators of 14-3-3σ/YAP1-induced gemcitabine resistance. More importantly, consistent with the prior findings that double knockdown of 14-3-3σ and YAP1 simultaneously did not further reduce gemcitabine resistance (Figure 4), simultaneous knockdown of 14-3-3σ and YAP1 fails to further reduce the expression of both RRM1 and RRM2 compared with single knockdowns (Figure 7D). Thus, RRM1 and RRM2 may be the key downstream mediators of 14-3-3σ/YAP1-induced gemcitabine resistance.

## DISCUSSION

While 14-3-3σ expression has been found to increase in cancer cells that have acquired drug resistant phenotype and contribute to the resistance, the detailed molecular mechanisms of its function in drug resistance remain elusive. Previously, it has been suggested that increased 14-3-3σ expression may cause resistance to drug-induced apoptosis [9], possibly by binding to and arresting cyclin B1 and CDC2 [21, 22] and pro-apoptotic proteins such as Bax and Bad [23, 24] in cytoplasm. Somatic knockout of 14-3-3σ in colon cancer cells has been shown to cause drug-induced mitotic catastrophe by reducing cellular ability to arrest in G2/M phase in response to DNA damage [21]. In this study, we identified a novel mechanism of 14-3-3σ-induced gemcitabine resistance in PDAC. As shown in Figure 7E, 14-3-3σ over-expression may promote YAP1 expression and interact with YAP1. The inter-dependent 14-3-3σ/YAP1 interaction contributes to acquired gemcitabine resistance by attenuating gemcitabine-induced caspase-8 activation and apoptosis, possibly via enhancing the expression of RRM1 and RRM2, which are well known mechanisms in gemcitabine resistance.

In this study, we found that 14-3-3σ not only interacts and binds to YAP1, it also regulates YAP1 expression. Because YAP1 mRNA level was also changed by 14-3-3σ, 14-3-3σ may regulate YAP1 transcription. Although transcriptional regulation of YAP1 expression has not yet been studied, analysis of human YAP1 promoter sequence shows potential binding site for p53, AP-1, and c-Jun (unpublished observations). Because MiaPaCa-2 cells carry an inactive mutant p53, 14-3-3σ unlikely regulates YAP1 transcription via p53 although 14-3-3σ has been shown to positively regulate p53 [25]. Whether other transcription factors such as AP-1 and c-Jun mediates 14-3-3σ regulation of YAP1 transcription remain to be determined. We also found that pYAP1 was dramatically altered by 14-3-3σ. While the increased pYAP1 may be due to the increased total YAP1 expression, it is also possible that 14-3-3σ binding to pYAP1 stabilizes pYAP1, which contributes to the increased level of total YAP1 although this may be unlikely since 14-3-3σ knockdown did not reduce ectopic GFP-YAP1 (Figure 3A).

14-3-3σ and YAP1 over-expression-induced gemcitabine resistance appears to be via protecting cells against gemcitabine-induced caspase-8 activation and apoptosis. This observation is consistent with a previous report that gemcitabine induces caspase-8 activation in H292 cells and enhances cell sensitivity to Fas-mediated cytotoxic activity [26]. Although it remains to be determined how gemcitabine induces caspase-8 activation, it has also been reported that gemcitabine induces apoptosis in non-small cell lung cancer (NSCLC) cells by increasing expression of functionally active Fas (CD95, APO-1) and up-regulating Fas ligand (FasL) [26, 27]. However, it remains to be determined if 14-3-3σ/YAP1 up-regulation contributes to inhibition of gemcitabine-induced caspase-8 activation by affecting Fas or FasL.

One of the major targets of gemcitabine is RRM1 and RRM2, which ensure sufficient supply of dNTP pool for DNA synthesis of proliferating cancer cells. Increased expression of RRM1 and RRM2 has been shown to contribute to acquired gemcitabine resistance [19, 20]. The finding that 14-3-3σ/YAP1 increases RRM1 and RRM2 expression is very intriguing while this finding suggests that RRM1 and RRM2 may be possible downstream mediators of 14-3-3σ/YAP1-induced gemcitabine resistance. However, it remains to be determined how 14-3-3σ/YAP1 up-regulates RRM1 and RRM2 expression. Because both 14-3-3σ and YAP1 exert their functions by binding to other proteins, it is tempting to speculate that the increased 14-3-3σ/YAP1 complex may increase RRM1 and RRM2 stability. Because YAP1 binds to other transcription factors and regulate gene transcription, 14-3-3σ/YAP1 complex may also regulate the transcription of RRM1 and RRM2. One such potential transcription factor is NF-κB, which has been shown to regulate RRM2 expression in gemcitabine-resistant human oral cancer KB cell line [28]. It has also been found that depleting glucose in culture media reduces RRM1 expression in PDAC cells [29]. However, it remains to be determined if 14-3-3σ/YAP1 participates in this regulation of RRM1 expression. Furthermore, CBL0137, a curaxin that inhibits chromatin remodeling complex FACT, sensitizes gemcitabine resistance in PDAC cells possibly by reducing RRM1 and RRM2 expression [30]. Interestingly, we previously observed that 14-3-3σ up-regulation in the gemcitabine resistant G3K cells was due to reduced gene methylation. It is, thus, of interest to determine if CBL0137 affects 14-3-3σ expression by inhibiting chromatin remodeling, which in turn down-regulates RRM expression and increases gemcitabine sensitivity.

In summary, 14-3-3σ may have multiple mechanisms of function in contributing to drug resistance. This multifaceted property may be derived from its activity being able to interact with multiple proteins. However, it remains to be determined if 14-3-3σ plays an important role in clinically acquired or intrinsic gemcitabine resistance in PDAC or clinical drug resistance in general although increased 14-3-3σ expression has been found in PDAC samples and appears to associate with poor prognosis of PDAC [6, 7]. It also remains to be determined if 14-3-3σ can be established as a potential target for drug discovery to sensitize drug-resistant human cancers in combinational chemotherapy. We are currently working toward addressing these questions.

## MATERIALS AND METHODS

### Materials

Metafectene Pro transfection reagent was obtained from Biontex. siRNAs targeting 14-3-3σ (sc-29590), YAP1 (sc-38637) were purchased from Santa Cruz Biotechnology. Antibodies against GFP (ab290), YAP1 (ab52771), pYAP1 (ab76252) were from Abcam. Antibodies against 14-3-3σ (05-632), RRM1 (MABE567) and ChIP Assay kit (17-295) were purchased from EMD Millipore. Antibody against RRM2 was generated in-house [31]. Lipofectamine, pcDNA3.1(+) plasmid, and G418 were from Invitrogen. RNeasy Mini kit and Qiagen Blood and Cell Culture DNA Kit were from Qiagen. The iScript™ cDNA synthesis kit and the SYBR Green PCR master mix were from Bio-Rad and Applied Biosystems, respectively. Gemcitabine were purchased from Besse Medical

### Cell lines, cultures, and transfections

The gemcitabine resistant human PDAC cell lines G500, G1K and G3K cells were generated by stepwise selection of MiaPaCa-2 cells using increasing concentrations of gemcitabine and cultured in DMEM medium supplemented with 10% fetal bovine serum and 2.5% horse serum in the presence of gemcitabine as previously described [8]. Human PDAC cell line ASPC-1 was from ATCC and cultured in RPMI medium supplemented with 10% fetal bovine serum. The cell lines were authenticated by analysis of tandem repeat sequences on September 17, 2013.

For transient knockdown or over-expression of target genes, cells were plated in a six-well plate at a density of 1.5-3×10^5^ cells/well and cultured overnight in complete medium. About 60-120 pmol siRNAs of target genes or control scrambled siRNAs, or 1-2 μg of over-expressing plasmid of target genes or vector control plasmid were diluted in serum-free Opti-MEM medium and then transiently transfected into cells using Metafectene Pro transfection reagent as previously described [32].

For stable transfection, the cDNA of 14-3-3σ gene was engineered into pcDNA3.1(+) and transfected into MiaPaCa-2 cells using Lipofectamine. Stable clones were selected using 1 mg/ml G418 as previously described [9, 22]. The stable clones were maintained in complete medium supplemented with 200 μg/ml G418.

Similarly, the stable shRNA knockdown was generated as previously described [9, 22]. Briefly, G3K cells were transfected with pSilencer-σ (14-3-3σ shRNA cloned into pSilencer 3.1-H1neo vector) or scrambled shRNA construct [9, 22] using Lipofectamine followed by selection with 1 mg/ml G418 for 2 weeks. Individual clones were tested for 14-3-3σ knockdown and positive clones were propagated and maintained in complete DMEM medium.

### Cell lysate preparation and western blot

Cultured cells were harvested, washed with PBS, and lysed in TNN-SDS buffer (50 mM Tris-HCl, pH 7.4, 150 mM NaCl, 0.5% Nonidet P-40, 50 mM NaF, 1 mM sodium orthovanadate, 1 mM dithiothreitol, 0.1% SDS, and 2 mM phenyl-methylsulfonyl fluoride) for 30 minutes at 4°C with constant agitation. The cell lysates were then sonicated briefly and followed by centrifugation (14,000×*g* at 4°C) for 15 minutes to remove insoluble materials. The protein concentrations of supernatants were measured by Bradford assay.

Cell lysates were separated by SDS-PAGE and transferred to a PVDF membrane followed by a 2-hr incubation in blocking solution (PBS-buffered saline containing 5% nonfat dried milk and 0.1% Tween 20) and a 2-hr incubation with primary antibodies. After extensive washes, immunoreactivity was detected with specific secondary antibodies conjugated to horseradish peroxidase. Signals were captured using ECL x-ray film.

### Survival and apoptosis assay

Survival assay was performed as previously described using MTT colorimetric assay [7, 33]. Briefly, cells were seeded in 96-well plate at 2000-3000 cells/well and cultured for 24 hrs followed by treatment with different doses of anticancer drugs and incubated continuously for 3 days followed by addition of MTT (5 mg/ml) to a final concentration of 0.5 mg/ml and incubation of the plates at 37°C for 4 hours. The OD_570nm_ and OD_630nm_ were measured using an automated plate reader and analyzed using GraphPad Prism software to generate fitted curve and IC_50_. Relative resistance factor (RRF) is calculated using the following formula: RRF=IC_50(test)_/IC_50(control)_. For apoptosis assay, photometric enzyme immunoassay using a Cell Death Detection ELISA Plus kit (Roche Diagnostics, Indianapolis, IN) was performed for quantitative in vitro determination of cytoplasmic histone-associated DNA fragments and apoptosis as previously described [34].

### Quantitative real-time RT-PCR

Quantitative RT-PCR was performed as described previously [35, 36]. Briefly, total RNA was extracted using RNeasy Mini Kit followed by reverse-transcription using iScript™ cDNA synthesis kit and quantitative PCR using the SYBR Green PCR master mix. The primer pairs used are: 5′-TAGGCGCTGTTCTTGCTCCAA-3′ (forward) and 5′-ACCAGTGGTTAGGTGCGCTCA-3′ (reverse) for 14-3-3σ; 5′-AAGAGCAGCGTGCCAGAGAT-3′ (forward) and 5′-ACACATCAAAGACCAGTCCTGATTAG-3′ (reverse) for RRM1; 5′-TCTGGCTTTCTTTGCAG CAA-3′ (forward) and 5′-CAGCGGGCTTCTGTAA TCTGA-3′ (reverse) for RRM2; 5′-CAGCAACTGC AGATGGAGAA-3′ (forward) and 5′-ACATCCCGGG AGAAGACACT-3′ (reverse) for YAP1; 5′-AAGGAC TCATGACCACAGTCCAT-3′ (forward) and 5′-CCAT CACGCCACAGTTTTC-3′ (reverse) for GAPDH.

### Immunofluorescence and confocal microscope imaging

1-2 × 10^5^ G3K cells were seeded on a glass coverslip in a six-well tissue culture plate. After the culture reaches confluence, the cells were washed 3 times with ice-cold PBS and fixed with acetone/methanol (1:1) at room temperature for 15 min and incubated with blocking solution (3% bovine serum albumin in PBS) for 1 h. The cells were then probed with primary antibody YAP1 (1:200) for 2 hrs followed by incubation with FITC-conjugated goat anti-rabbit IgG F(ab')_2_fragment (Sigma) (1:1000 dilution) for 30 min. After being washed 3 times with blocking solution, the cells were re-probed with another primary antibody 14-3-3σ (1:50) for 2 hrs followed by incubation with Alexa Fluor 647 dye (Life Technologies) for additional 30 min. Then, after being washed 3 times, the cell nucleus was counterstained with DAPI (25 μg/ml) for 20 min. The coverslips were then mounted on the slides before viewing with Olympus 2 confocal microscope. The laser excitation lines are as follows: 405 nM for DAPI, 488 nM for FITC, and 635 nM for Alexa Fluor 647. The image was then virtualized by Olympus Fluoview Ver.3.0 viewer (FV10-ASW 3.0 viewer).

### Immunoprecipitation assay

Immunoprecipitation was performed as previously described [37]. Briefly, 1 mg of cell lysates were first pre-cleaned by incubation with 1 μg of normal mouse IgG at 4°C for 1 h, then mixed with 150 μL of protein G agarose beads (50% slurry) and incubated at 4°C for 2 hrs followed by centrifugation at 500× *g* for 5 min. The cleared supernatants were split into two equal parts incubated with either normal mouse IgG (as a negative control) or incubated with primary antibodies (anti-Flag, anti-YAP1, anti-pYAP1, or anti-GFP) at 4°C for 3 h, then each part was mixed with 50 μL of protein G agarose beads. After overnight incubation at 4°C, the reaction was centrifuged to collect precipitates which were then washed five times with lysis buffer (50 mM Tris-HCl, pH 7.4, 150 mM NaCl, 1 mM EDTA, 1% Triton X-100) before being subjected to SDS-PAGE analysis for Western blot analysis.
